# Microbiome-Metabolomics Analysis of the Impacts of *Cryptosporidium muris* Infection in BALB/C Mice

**DOI:** 10.1128/spectrum.02175-22

**Published:** 2022-12-19

**Authors:** Luyang Wang, Letian Cao, Yankai Chang, Yin Fu, Yuexin Wang, Kaihui Zhang, Sumei Zhang, Longxian Zhang

**Affiliations:** a College of Veterinary Medicine, Henan Agricultural University, Zhengzhou, China; b International Joint Research Laboratory for Zoonotic Diseases of Henan, Zhengzhou, China; c Key Laboratory of Quality and Safety Control of Poultry Products (Zhengzhou), Ministry of Agriculture and Rural Affairs, Zhengzhou, China; Institut Pasteur

**Keywords:** *Cryptosporidium*, gut microbiota, metabolites, short-chain fatty acid, bile acid

## Abstract

Cryptosporidium is a leading cause of diarrheal disease and mortality in young children worldwide. Cryptosporidium invades small intestinal epithelial cells and forms a unique intracellular niche, a process that may alter gut microbes and their production metabolites. However, the mechanism of interactions between gut microbes, metabolites, and parasites is poorly understood. Here, we first characterized the impacts of Cryptosporidium infection on gut microbiota using a microbiome-to-metabolome association study. BALB/c mice were gavaged with Cryptosporidium muris, and fecal samples were collected at 0, 7, 14, 21, and 28 days postinfection (dpi) to observe changes in the intestinal microbes of the body during parasite infection. The infected group had a significantly increased relative abundance of bacterial taxa, such as *Lachnospiraceae* and Prevotella (*P* < 0.05), associated with the biosynthesis of short-chain fatty acids (SCFAs). Metabolites related to the metabolic pathways, steroid hormone biosynthesis, and biosynthesis of unsaturated fatty acids pathway were upregulated at 7 dpi, indicating that related metabolites in the biosynthesis of unsaturated fatty acids may be essential for C. muris reproduction *in vivo*. The metabolites involved in metabolic pathways, bile secretion, and primary bile acid biosynthesis were upregulated at 14 dpi, and we speculate that these metabolites may be critical to the growth and development of Cryptosporidium oocysts in the host. Correlation analysis revealed that *Firmicutes* bacteria are significantly associated with α-linolenic acid metabolism pathways (*P*< 0.05). The gut microbiota changes dynamically, and the metabolites involved in fatty acid and bile acid biosynthesis may play important roles during cryptosporidiosis. Details of the gut microbiota and the metabolome after infection with Cryptosporidium may aid in the discovery of specific diagnostic markers and help us understand the changes in parasite metabolic pathways.

**IMPORTANCE** Cryptosporidiosis is a gastrointestinal disease in humans and animals caused by the protozoan parasite Cryptosporidium. Control and treatment of the disease is challenging due to the lack of sensitive diagnostic tools and effective chemotherapy. The dynamics of gut microbiota and metabolites during Cryptosporidium infection may be the key to finding drugs and targets for parasite infection control. Our results indicate that C. muris infection can disrupt gut microbiota and metabolites, resulting in decreased bacterial abundance at the parasitic site. Unsaturated fatty acid pathway biosynthesis-related metabolites are significantly elevated at the patent period. Interestingly, the metabolite pathway that significantly elevated during peak parasite growth was bile acid, the metabolites of which may be important for the circulation of infection of Cryptosporidium oocysts in the host. The enhancing effects of short-chain fatty acid and bile acid metabolism on the growth and development of Cryptosporidium proposed in this study may provide a theoretical basis for future research on novel drugs and vaccines against this intestinal parasite.

## INTRODUCTION

Cryptosporidium spp. are apicomplexan parasites that are among the top three causes of diarrhea in children under 2 years of age (1). Cryptosporidium muris was the first species of Cryptosporidium discovered. It is a zoonotic parasite discovered by Tyzzer in the stomach glands of laboratory mice in 1907 (2, 3). The life cycle of C. muris is similar to other Cryptosporidium species, but the infection site of C. muris is the stomach rather than the intestinal mucosal epithelial cells (4, 5). The diarrhea induced by Cryptosporidium is caused by significant mucosal inflammation, reduced absorption surface area, and malabsorption (6). The only U.S. Food and Drug Administration (FDA)-approved anti-Cryptosporidium drug, nitazoxanide, has limited efficacy and is not approved for use in immunocompromised patients and children under the age of 2 years (7). Thus, there is interest in new treatments and prevention strategies.

Many studies have shown that the gut microbiota is critical for intestinal homeostasis and that various intestinal and extraintestinal diseases are associated with dysbiosis (8, 9). Individuals are more susceptible to opportunistic pathogens, such as Cryptosporidium, when their gut microbiota is dysregulated (10–12). This was also demonstrated in a previous study of Cryptosporidium infection of protein-deficient mice to mimic some of the metabolic changes in malnourished children (13). Cryptosporidium infection can cause changes in metabolites in organisms, and these metabolites may be used as nutrients to supplement streamlined metabolism of parasites (14–16). Microbiome-metabolome correlation studies have helped reveal the interplay between gut microbes, metabolites, and host health (17). However, the molecular and biochemical mechanisms responsible for these broad effects are unknown. Therefore, the study of the changes in the gut microbiota and metabolome of mice after C. muris infection increases understanding of the host-parasite-microbiome interactions and provides a basis for the discovery of specific diagnostic markers and the analysis of parasite metabolic pathways.

In this study, Illumina high-throughput sequencing was used to determine the changes in the microbiome of mice before and after C. muris infection and at different infection stages. Liquid chromatography-mass spectrometry (LC-MS) was used to detect the changes of metabolites in mice at different periods before and after C. muris infection. Changes in the microbiome and metabolites of mice were then analyzed. This lays the foundation for further research on the gut microbiota and the regulation mechanism of C. muris infection and also provides a reference for the association study of gut microbiota and metabolomics for other protozoa that infect the host.

## RESULTS

### Cryptosporidium infection leads to altered fecal-microbiota structure.

**FIG 1:**
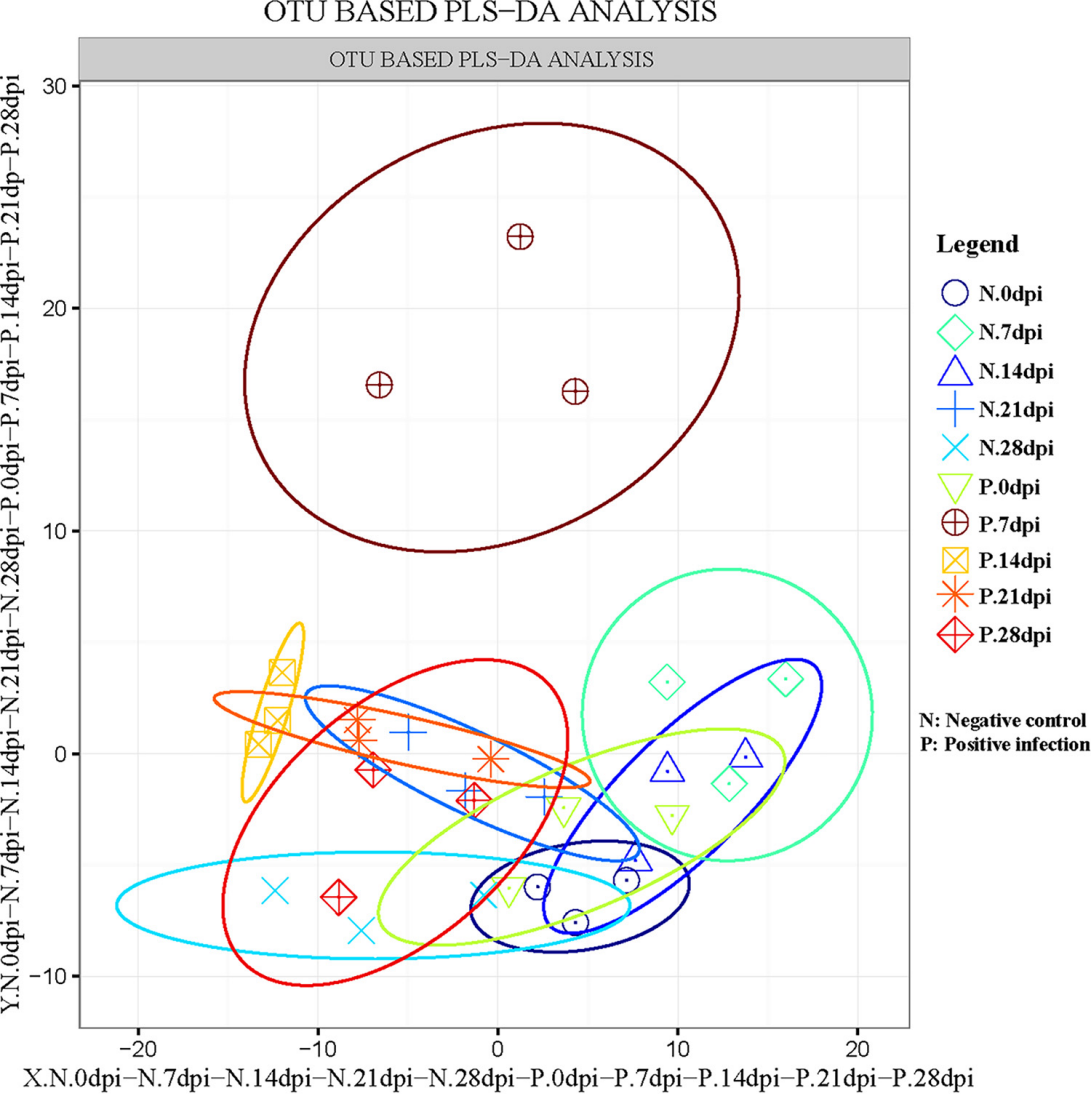
Operational taxonomic unit (OTU) partial least squares discriminant analysis (PLS-DA) of the negative control groups (N) and positive infection groups (P). The abscissa and the ordinate, respectively, represent the suspected influencing factors of the differences in the microbial composition of each group The scale is the relative distance and has no practical significance. dpi, days postinfection.

The appetites and attitudes of all animals in both the test and control groups were normal during the experiment. Histological analysis illustrated dense colonization of the gastric glandular parts at 14 days postinfection (dpi). Scanning electron microscopy (SEM) revealed that the stomach contained gastric pits filled with C. muris that adhered to the surface of gastric mucosa gland epithelial cells, causing them to deform, swell, and become disordered (Fig. S1 and S2). The species accumulation curve (Fig. S3A) and rarefaction curve (Fig. S3B) displayed good sample sizes and sequencing depth for analysis of the fecal microbiota. To estimate the effect of mice infected with C. muris on the gut microbial populations, the richness of operational taxonomic units (OTUs) between infected groups and control groups at different dpi was compared using partial least squares discriminant analysis (PLS-DA). During the comparisons at 7 and 14 dpi, there were larger between-group differences between the infected and uninfected groups (Fig. 1).

**FIG 2:**
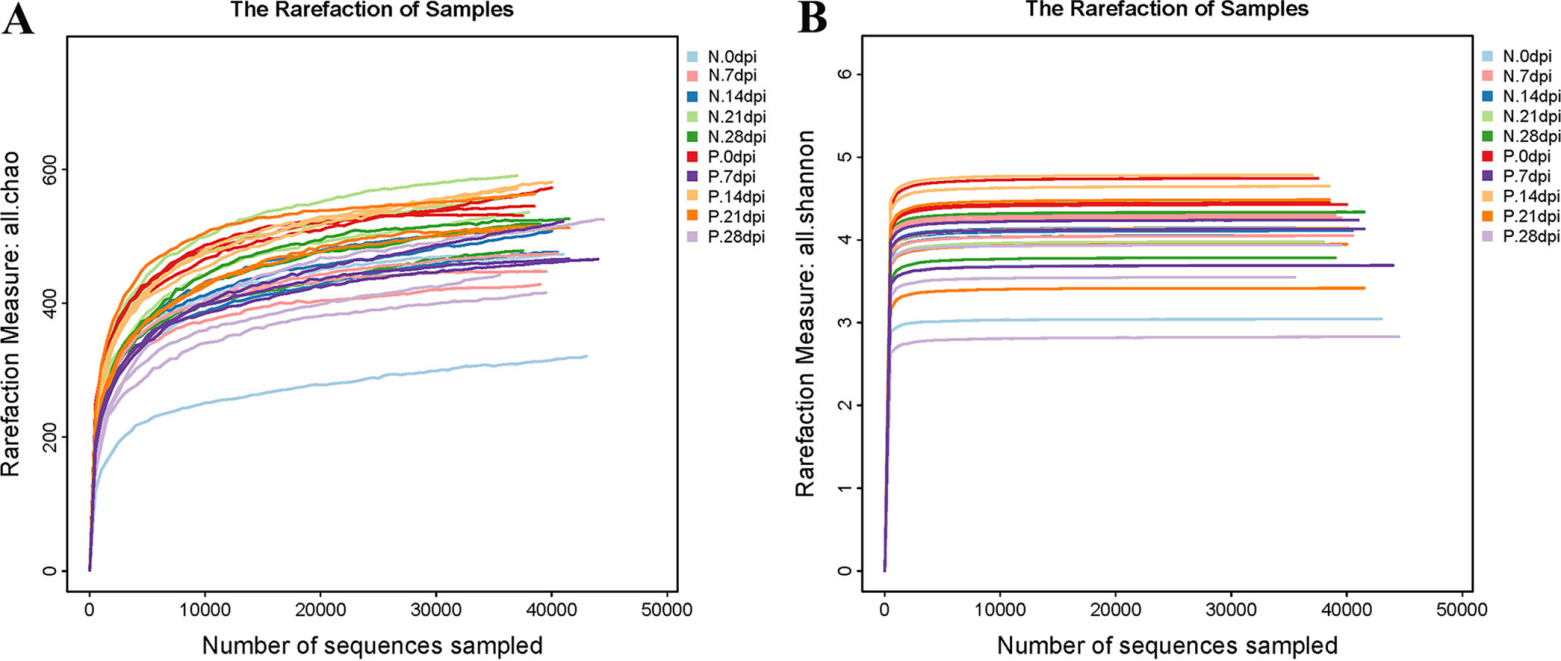
Chao indices (A) and Shannon-index curves (B) of all 30 fecal samples. N, negative-control group; P, positive-infection group.

**FIG 3:**
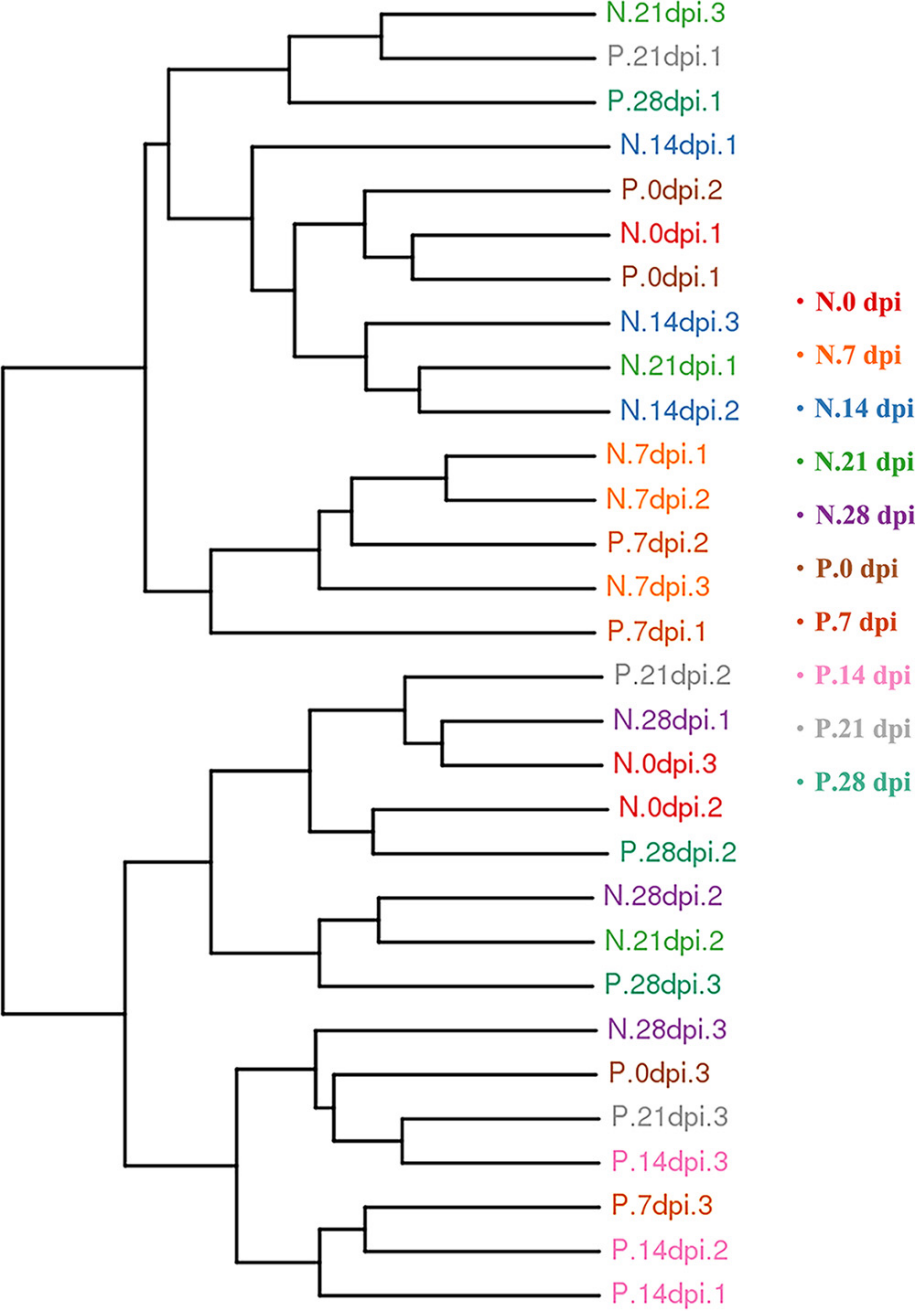
Differences in β-diversity (unweighted pair group method with arithmetic mean [UPGMA]-weighted UniFrac distance) between infected groups and control groups at different dpi. The fecal samples are clustered according to similarity between each other; the shorter the branch length between samples, the more similar the two fecal samples. N, negative-control group; P, positive-infection group.

The Chao indices and Shannon index curves demonstrated that most of the diversity was captured (Fig. 2). In the α-diversity analysis, the ACE and Chao indices reflected the community richness, and the Shannon and Simpson indices were used to assess the microbiota diversity. The results are shown in Table S1. The values showed no obvious changes in the microbiota richness and diversity at 0, 7, 21, and 28 days after C. muris infection. Conversely, significantly increased ACE index (*P* < 0.01) and Chao index (*P* < 0.01) at 14 days after C. muris infection indicated that the fecal-microbiota richness was increased during this period. Overall, these data suggested that mice infected with C. muris had higher microbiota richness at the peak of oocysts shedding (14 dpi) (5). β-Diversity analysis was used to confirm the differences and similarities in gut microbial community composition between groups. Hierarchical clustering analysis based on the weighted UniFrac unweighted pair group method with arithmetic mean (UPGMA) confirmed that the gut microbiota of the P.14 dpi group showed high similarity and different clustering of microbial composition for each group during the different dpi (Fig. 3).

### Changes in bacterial community structure during Cryptosporidium infection.

**FIG 4:**
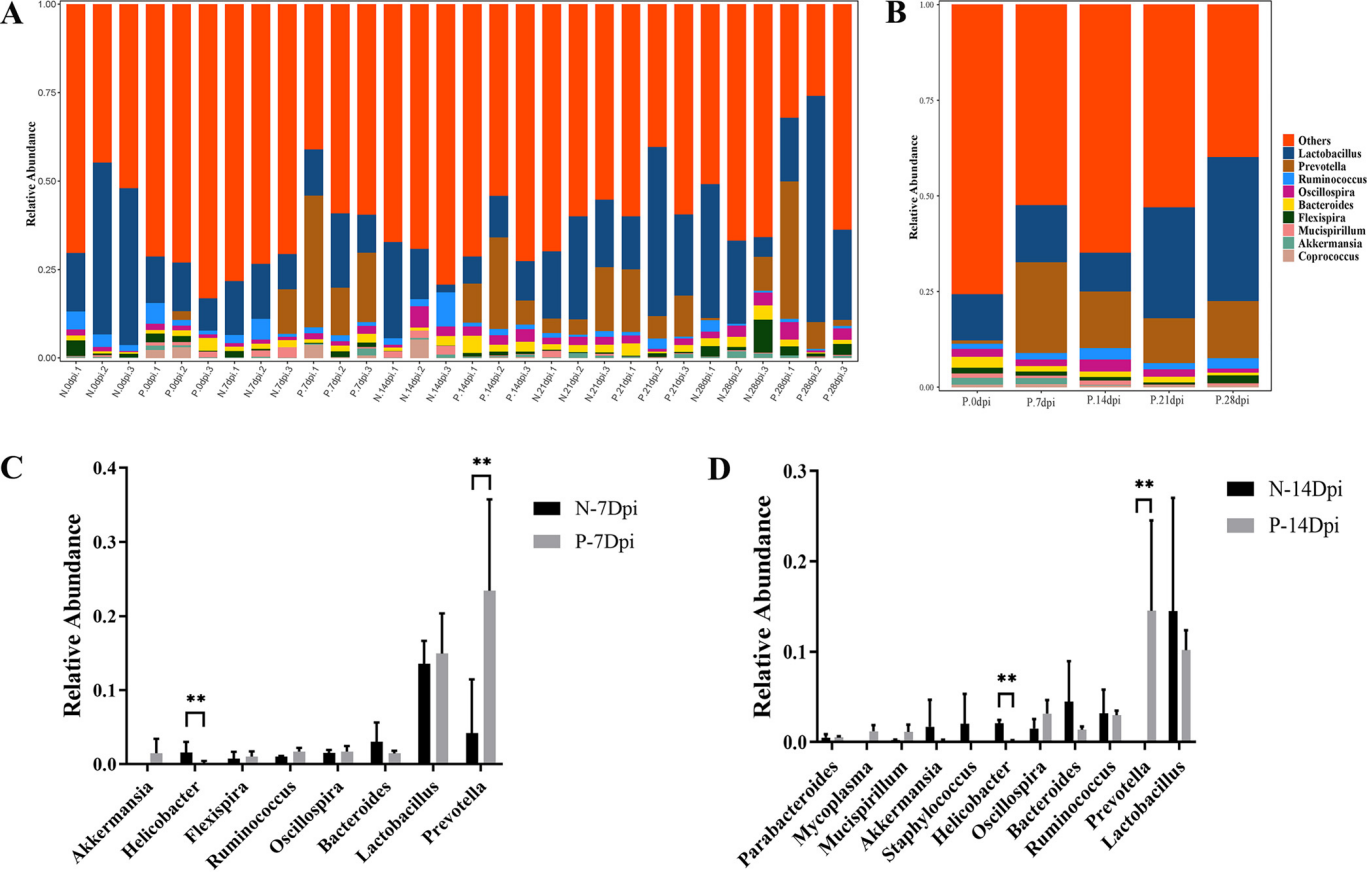
Differences in microbial composition of the microbiota at the genus level in control mice fecal (N) and C. muris-infected mice fecal (P). (A) Taxonomic distributions of the gut microbiota at the genus level in all samples. (B) Average gut microbial composition of the genus level in five infected groups. The abscissa shows the sample name, and the ordinate shows the relative abundance of the annotated species. The unannotated species of this classification level are classified as unclassified species with abundance below 0.5% in all samples are combined into Others. (C, D) Relative abundance of the gut microbial community between control versus infected groups at genus level. (C) At 7 dpi, control versus infected groups. (D) At 14 dpi, control versus infected groups. The figure shows the difference ratio of functional abundance in 95% confidence interval, the asterisks represents the significant difference of *P* < 0.05 (*) and *P* < 0.01 (**), respectively; the values were determined by *t* test.

The relative abundances at the phylum level revealed that *Firmicutes* levels in the infected group were significantly higher than those in the control group (61.09% versus 40.00%, *P* < 0.01), whereas the level of *Bacteroidetes* was significantly lower than that in the control group at 14 dpi (32.36% versus 52.72, *P* < 0.01) and showed dynamic changes during infection. Microbiota showed no significant difference in relative abundance between control and infected groups at other time points (Fig. S4A). Compared with the control groups, the following families were significantly increased in the infected groups: *Desulfovibrionaceae* (0.23% versus 1.69%, *P* < 0.05) and *Lachnospiraceae* (8.80 versus 20.51%, *P* < 0.05). Conversely, *Helicobacteraceae* (2.16% versus 1.09%, *P* < 0.05) and S24-7 (41.61% versus 9.81%, *P* < 0.05) were significantly reduced in the infected groups compared to the control groups at 14 dpi (Fig. S4B). At the genus level, Prevotella was significantly increased in the infected groups compared to the control groups at 7 dpi (23.42% versus 4.12%, *P* < 0.01) and at 14 dpi (14.55% versus 0.00%, *P* < 0.01), whereas the level of Helicobacter was significantly lower than that in the control group at 7 dpi (0.16% versus 1.56%, *P* < 0.01) and at 14 dpi (0.17% versus 2.09%, *P* < 0.01) (Fig. 4).

### Changes in metabolites associated with Cryptosporidium infection.

**FIG 5:**
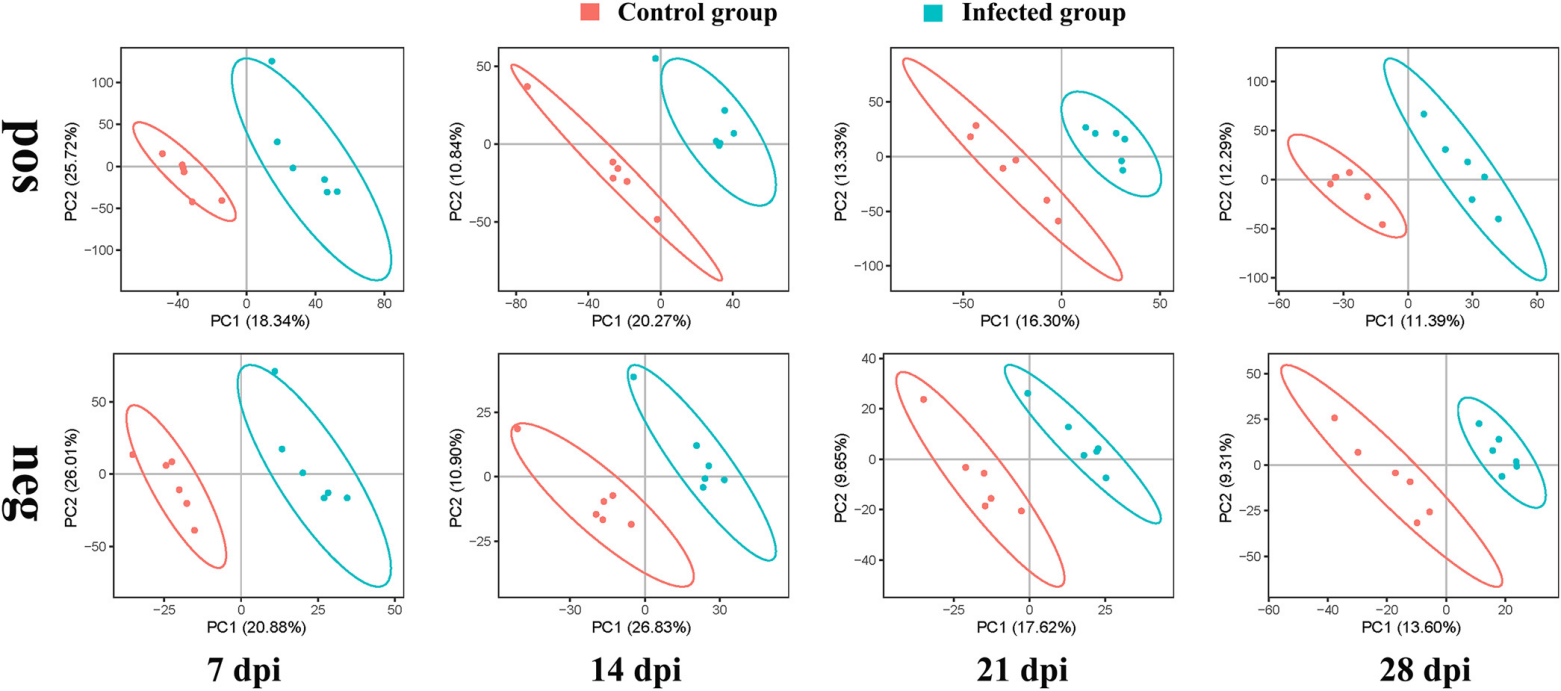
Unsupervised multivariate analysis of the global metabolic changes in fecal samples from control and infected group mice. Shown are the PLS-DA score plots of the first two principal components (PC1 and PC2) indicating the effect of infection on the metabolic profile of mouse feces at 7, 14, 21, and 28 dpi. Distinct metabolic differences were observed between the infected and control mouse groups. “pos” and “neg” indicate positive ion mode and negative ion mode, respectively.

A total of 2,267 compounds were detected in the negative ion mode, and 1,176 metabolites were putatively identified, whereas 7,507 compounds were detected in the positive ion mode with 3,964 metabolites putatively identified. PLS-DA analysis was performed to identify the differences in the metabolic profile between mice infected with C. muris and noninfected mice. Fig. 5 shows that in both positive and negative ion modes, the metabolites of infected and control mice were clustered in separate groups. As the infection resolved at 21 and 28 dpi, the differences and separation between infected and noninfected groups decreased. This indicates that the metabolic patterns of the infected groups were distinct from those of noninfected mice, especially during the earlier exposure periods (7 and 14 dpi).

**FIG 6:**
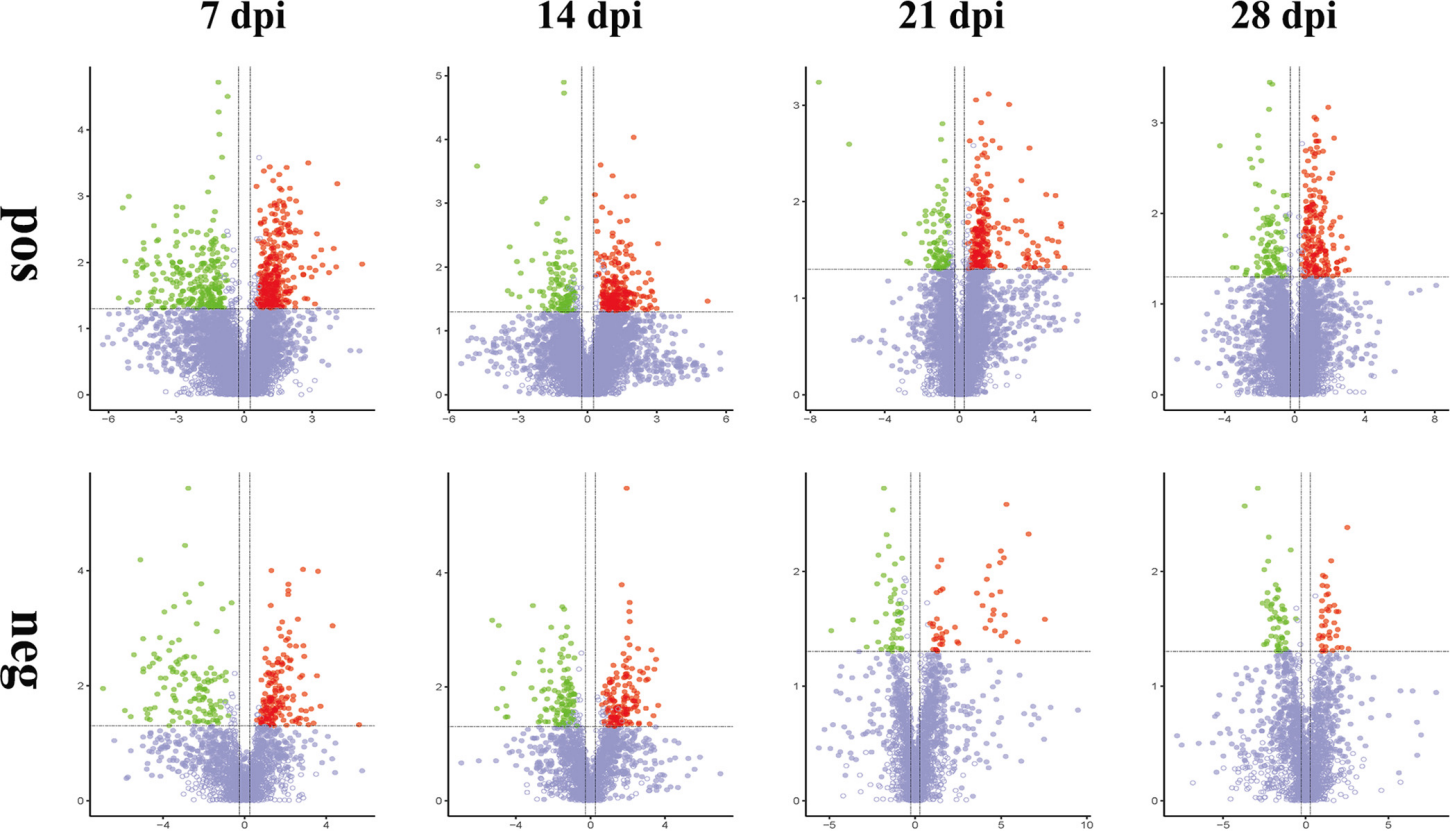
Volcano plots of the metabolic differences between infected and noninfected mice fecal samples at 7, 14, 21, and 28 dpi. The log_2_-fold change is shown on the *x* axis. Statistical significance displayed by −log_10_ (*P* value) is shown from 0 to 5 on the *y* axis. Metabolites having fold changes of >1.2 and *P* < 0.05 (Student’s *t* test) are represented by red dots. Metabolites having fold changes of <0.83 and *P* < 0.05 (Student’s *t* test) are denoted by green dots. Metabolites with no difference are marked in purple-gray dots. Fold changes of the infected samples relative to the untreated control are based on the mean values of six biological replicates per group. “pos” and “neg” indicate positive ion mode and negative ion mode, respectively.

**FIG 7:**
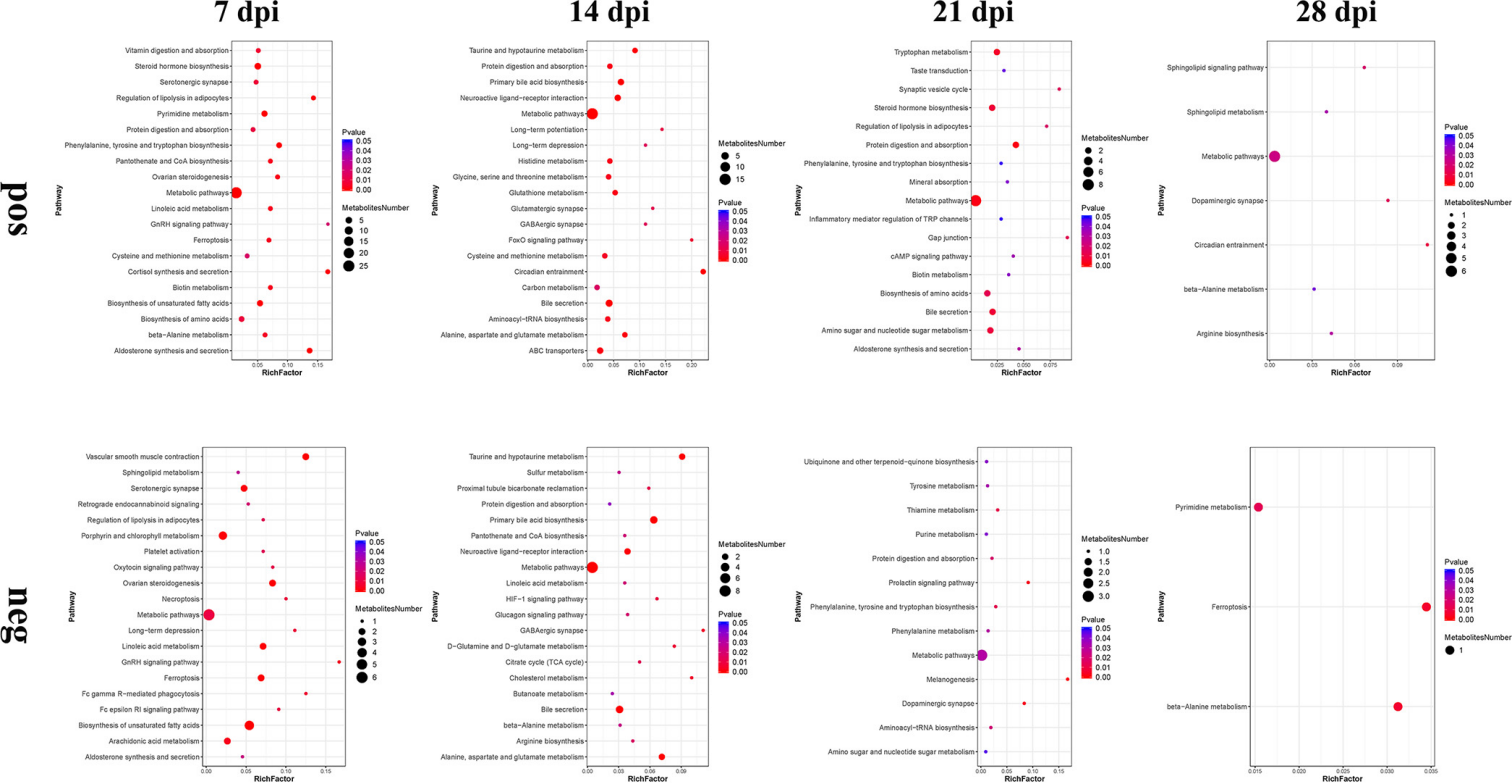
Bubble plot of pathways significantly enriched for differential metabolites between infected and noninfected mice fecal at 7, 14, 21, and 28 dpi. The *x* axis rich factor is the number of differential metabolites annotated to the pathway divided by all the identified metabolites annotated to the pathway. The larger the value, the greater the proportion of differential metabolites annotated to the pathway. Dot size represents the number of differential metabolites annotated to that pathway. “pos” and “neg” indicate positive ion mode and negative ion mode, respectively.

To identify the temporal differences between the feces of infected and noninfected mice, the differences in the metabolites between C. muris-infected groups and noninfected groups were analyzed at 7, 14, 21, and 28 dpi. Table S2 shows that the fecal metabolomic alterations were distinct between infected groups and control groups. The number of upregulated metabolites and downregulated metabolites at 7 dpi was greater than that at other time points. The volcano plots of these metabolites are shown in Fig. 6. The details of the differentially abundant metabolites that were mapped to KEGG pathways are shown in Table S3. At 7 dpi, the differentially abundant metabolites are potentially involved in 31 and 22 differential metabolic pathways in positive (pos) and negative (neg) mode, respectively. At 14 dpi, the differentially abundant metabolites are potentially involved in 31 and 26 differential metabolic pathways in pos and neg mode, respectively. at 21 dpi, the differentially abundant metabolites are potentially involved in 17 and 13 differential metabolic pathways in pos and neg mode, respectively. at 28 dpi, the differentially abundant metabolites are potentially involved in seven and three differential metabolic pathways in pos and neg mode, respectively. Bubble plots were drawn for pathways significantly enriched for differential metabolites, as shown in Fig. 7. At 7 dpi, the differential metabolites are mainly involved in metabolic pathways, steroid hormone biosynthesis, and biosynthesis of unsaturated fatty acids. At 14 dpi, the differential metabolites are mainly involved in metabolic pathways, bile secretion, and primary bile acid biosynthesis.

### Correlation between fecal microbiota and metabolic pathway.

**FIG 8:**
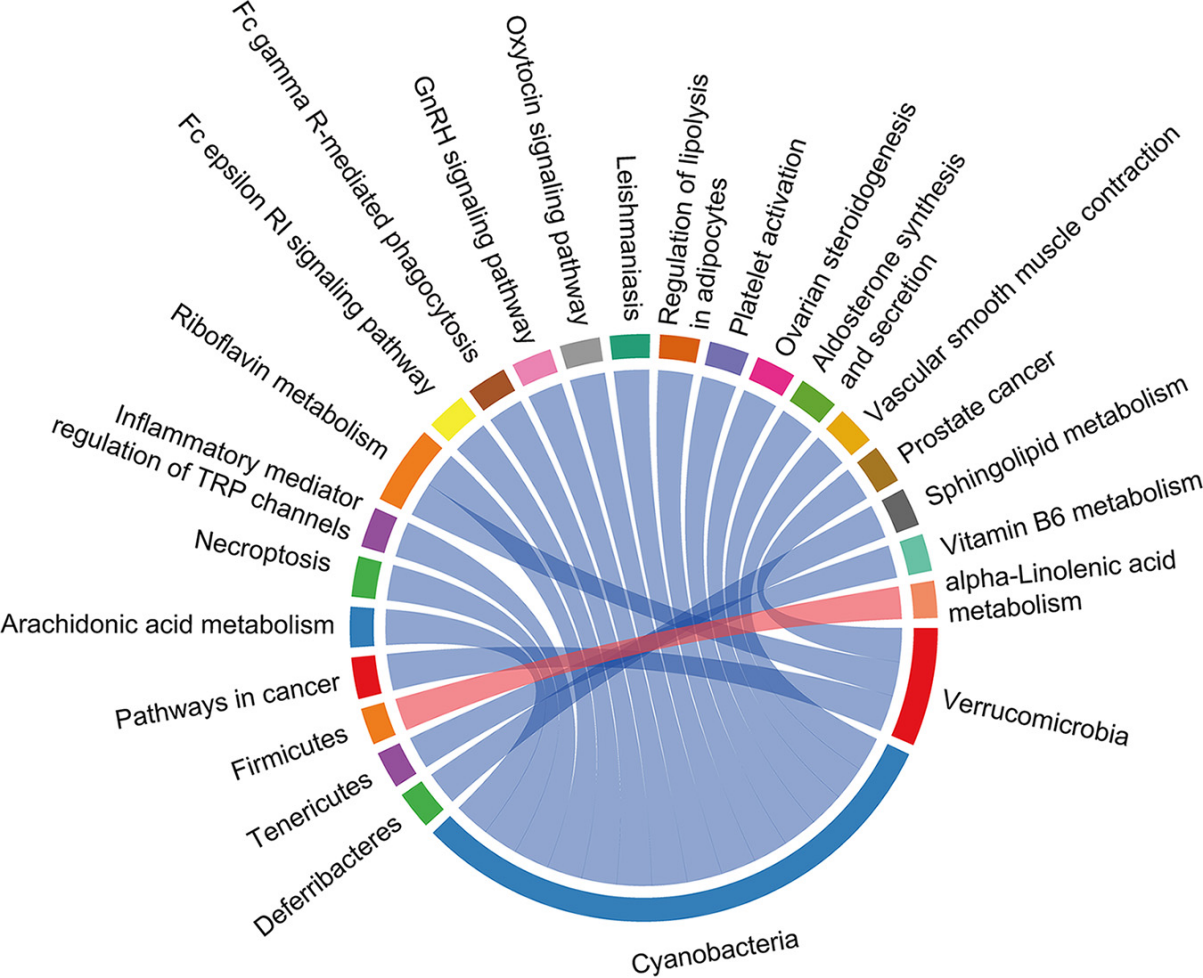
Correlation network analysis among the perturbed gut-bacterial (at the genera level) and differential metabolic pathways. The absolute value of Spearman’s rank correlation coefficients is the largest, and the *P* value < 0.05. Each node represents a metabolic pathway or microbial group, the red arc between nodes represents a negative correlation, and the blue arc represents a positive correlation.

The 16S rRNA gene sequencing revealed that C. muris infection altered the gut microbiota composition of mice, and metabolomics analysis demonstrated that exposure to C. muris perturbed the metabolic profiles. We also examined the functional correlations between the disturbed gut microbiota (at the phylum level) and the pathway through a correlation analysis based on the top 20 relationship pairs with the largest absolute value of Spearman’s correlation coefficient and *P* < 0.05 (Fig. 8). Correlation analysis revealed that *Firmicutes* with significant differences had a positive correlation with α-linolenic acid metabolism pathways (*P* < 0.05), while the *Tenericutes* and *Deferribacteres* were negatively correlated with vitamin B_6_ metabolism and sphingolipid metabolism, respectively (Fig. 8).

## DISCUSSION

The gut microbiota in the body and the external environment will maintain a dynamic balance. Factors such as diet, disease, drugs, and parasites can affect the gut microbiota of mice, resulting in dysbiosis (18, 19). There are an increasing number of reports on the changes in gut microbiota caused by Cryptosporidium infection, but the changes in the intestinal microbes of the body during Cryptosporidium infection and the underlying mechanism are not known (11, 16, 20). The goal of this study was to determine the changes in the gut microbiota and metabolites of BALB/c mice in different periods before and after C. muris infection using 16S rRNA sequencing and liquid chromatography-tandem mass spectrometry (LC-MS/MS).

Bacteria such as *Lachnospiraceae* and *Desulfovibrionaceae* increased in abundance during C. muris infection, especially at 14 dpi. Interestingly, previous studies have shown that the number of shed oocysts peaks on the second week after C. muris infection in mice (5, 21). *Lachnospiraceae* contribute toward to pyruvate metabolism to drive SCFA biosynthesis (22). Additionally, the role of the gut microbiome in the production of short-chain fatty acids (SCFAs) is now better understood (23, 24), especially in the fight against parasitic infections (25, 26). *Desulfovibrionaceae* is a sulfate-reducing bacterium that can decompose organic acids, short-chain fatty acids, amino acids, and other nutrients. These bacteria can reduce sulfate to produce hydrogen sulfide (H_2_S). H_2_S can be toxic to aerobic organisms that possess the mitochondrial cytochrome *c* oxidase enzyme (27). H_2_S is also proinflammatory, which can promote the development of irritable bowel disease (IBD) and colorectal cancer by disrupting cellular integrity (28). A moderate amount of hydrogen sulfide produced by *Desulfovibrionaceae* was shown to contribute to the growth of *Entamoeba* (29). We observed that the abundance of *Helicobacteraceae* and S24-7 was significantly reduced during C. muris infection. *Helicobacteraceae* bacteria mainly cause gastrointestinal diseases, and the decrease of *Helicobacteraceae* in the infected group was mainly due to the decrease of Helicobacter. However, a recent study showed that the relative abundance of Helicobacter increased with the increased level of increasing Cryptosporidium parvum infection (10). This may be because Helicobacter in the stomach mainly colonizes the deep gastric pits and gastric mucosa (30), while C. muris can cause pathological changes in the gastric mucosa (5). The invasion of foreign C. muris may disrupt the habitat of the indigenous microbiota, and this explains the decline in their reduced abundance, although further verification is needed. Loss of gastrointestinal integrity and increased motility following C. muris infection may account for the greatly reduced abundance of some major anaerobic commensal bacteria such as Bacteroides S24-7. The would be consistent with results of Cystoisospora suis infection (31). Other bacterial genera such as Prevotella were also disturbed in our study. Prevotella is associated with intestinal immunity and resistance to C. parvum infection (10) and is also associated with SCFA biosynthesis (32).

LC-MS/MS-based metabolomics and multivariate statistical analyses revealed a clear difference between the infected groups and the control groups. This difference gradually converged as infection progressed and may have resulted from homeostatic recovery when the infection progressed to the later stage. Lipids play an important role in the pathogenic mechanism of Cryptosporidium infection. Fatty acid biosynthesis and availability can limit parasite growth and development (16, 33). Medium- or long-chain saturated fatty acids can inhibit C. parvum growth by negatively affecting its streamlined metabolism, conversely, long-chain unsaturated fatty acids can enhance C. parvum invasion by regulating membrane fluidity (24). In the present study, the metabolites involved in the biosynthesis of unsaturated fatty acids (adrenic acid, eicosapentanoic acid, arachidonic acid, and 8*z*,11*z*,14*z*-eicosatrienoic acid) were upregulated in the infected group at 7 dpi. These metabolites are all long-chain unsaturated fatty acids, which may be essential for C. muris invasion. In addition, the bile acid metabolism pathway is also a key metabolic pathway altered by eicosapentanoic acid (34). We found that indole was also upregulated in the feces of infected mice, while high concentrations of indole have a certain promotion effect on the resistance to Cryptosporidium infection. Furthermore, indole concentrations in the gut at the time of Cryptosporidium exposure have proven to be an important biomarker (35). The metabolites involved in bile acid biosynthesis (taurine, taurochenodeoxycholic acid, deoxycholate, and cholic acid) were upregulated in the infected groups at 14 dpi. Similarly, a neonatal mouse infection model study demonstrated that Giardia lamblia infection can induce bile secretion and use the bile constituent as a substrate for parasite growth (36). In addition, bile salts have been identified as an important trigger for excystation of Cryptosporidium oocysts (37, 38). Therefore, we speculate that the metabolites involved in bile acid biosynthesis may also play a certain role in the growth and development of Cryptosporidium. In the study of cryptosporidiosis, upregulated bile acid biosynthesis in mouse feces is a novel feature that requires further description through targeted metabolomics studies.

In this study, we collected fecal samples from mice before and after C. muris infection at different times instead of using blood samples to investigate gut microbiota and metabolites. Molecules in the fecal cloud can reveal the most essential link between the gut microbiota and its metabolites (39). Our gut microbiota-to-metabolome association study demonstrated that the abundance of *Firmicutes* with significant differences was positively correlated with α-linolenic acid metabolism pathways. Butyrate, the major metabolite of *Firmicutes*, can regulate inflammatory cytokine activity, especially in the gut of a host with colitis or IBD (40, 41). Additionally, the role of SCFAs against Cryptosporidium infection has been highlighted (16). However, further studies evaluating SCFAs against Cryptosporidium are needed to discover detailed mechanisms.

### Conclusions.

We investigated the effects of C. muris infection on the fecal gut microbial composition by 16S rRNA genes sequencing and on the fecal metabolome using untargeted metabolomics approaches. This mouse study increases our understanding of the fecal biochemical composition during the C. muris infection process. The results suggest that during C. muris infection, the gut microbiota changes dynamically. The metabolites involved in fatty acid and bile acid biosynthesis may play important roles during cryptosporidiosis. However, the role of bile acid biosynthesis in resistance to cryptosporidiosis is unclear. These alterations reflect pervasive interactions between the host, pathogen, microbiota, and metabolites that occur during parasite colonization. The findings may provide a basis for the development of new anti-Cryptosporidium drugs and a novel vaccine.

## MATERIALS AND METHODS

### Cryptosporidium infection and sample collection.

We purchased 40 BALB/c mice aged 3 to 4 weeks from Henan Animal Experiment Center (Henan, China) and randomly divided them into 2 groups: the positive-infection group (P) and the negative-control group (N). The 40 mice were housed with 10 animals per cage and were adaptively fed for 7 days under the same environmental conditions to minimize the difference in the composition of the gut microbiota between individuals. All mice were provided nonmedicated feed and water *ad libitum* throughout the experiment.

The C. muris used originally isolated from two Bactrian camels (4 years old) and purified from fecal material by the water/ether concentration method and sucrose density gradient centrifugation, as previously described (5, 42, 43). The fresh C. muris oocysts were stored at 4°C in 2.5% potassium dichromate until use. Before experiments, oocysts were treated with 10% Clorox on ice for 10 min and washed three times with sterile phosphate-buffered saline (PBS). The 20 mice in the positive-infection group were gavaged with 3 × 10^6^ C. muris oocysts suspended in 200 μL distilled water, and the mice in the negative-control group were inoculated with the same volume of distilled water. The mice were free to access rodent chow and water throughout the trial. Body weight and food/water intake were monitored. Five mice in the N and P groups were sacrificed at 14 dpi. The oocysts were examined by light microscopy of modified acid-fast stained gastric tissue. Stomach tissue was obtained for histology and SEM observation. According to the previous report (5), 10 mice were randomly selected from each group at 0, 7 (C. muris oocysts were first detected in feces), 14 (peaking first of oocyst shedding), 21 (peaking second of oocyst shedding), and 28 (spontaneously clear the infection) dpi, and the abdomen of each mouse was gently rubbed for the collection of fecal samples. Although there are some differences in different infection tests, the overall infection dynamics were regularized. After collection of fecal samples, the mice were put back into their cages to continue feeding. Three eligible samples were randomly selected from the N and P groups at each time point for the subsequent gut microbiota analyses, and six eligible samples (including three samples for gut microbiota analyses) were randomly selected from the N and P groups at each time point for the subsequent metabolome analyses. The fecal samples were stored at −80°C until analysis.

### Fecal microbiota: 16S rRNA sequencing.

Genomic DNA extraction was performed on each stool sample using an E.Z.N.A. stool DNA kit (Omega BIO-TEK, Inc., Norcross, GA, USA). The concentration and quality of the extracted DNA samples were determined by Nano Drop 2000. Three qualified DNAs were selected from each group for use with the universal forward primer (5′-ACTCCTRCGGGAGGCAGCAG-3′) and reverse primer (5′-GGACTACCVGGGTATCTAAT-3′) to amplify the 16S rRNA genes of all bacteria in the V3-V4 region by PCR (PCR). We used Agencourt AMPure XP magnetic beads to purify the PCR amplification products, dissolved them in elution buffer, labeled them, and completed the library construction. An Agilent 2100 Bioanalyzer (Agilent, CA, USA) was used to detect the fragment range and concentration of the library. According to the size of the inserted fragment, the qualified library was sequenced on the HiSeq platform at the Beijing Genomics Institute Company, Ltd. (Shenzhen, China).

### Bioinformatics and statistical analyses.

The original DNA data were processed to obtain clean data, and FLASH software (Fast Length Adjustment of Short Reads, version 1.2.11) was used to assemble the paired reads obtained by pair-end sequencing into a sequence by using the overlap relationship to obtain the hypervariable region tags. We used UPARSE (version 7.0.1090) to perform clustering at 97% similarity to obtain the representative sequence of OTU, and used UCHIME (version 4.2.40) to remove the chimera generated by PCR amplification from the representative sequence of OTU. The representative sequence of each OTU was aligned against the Silva, Greengene, and UNITE databases for taxonomic analysis. The annotation results are filtered as follows: (1) remove OTUs without annotation results; and (2) remove species with annotation results that do not belong to the analysis project. The remaining OTUs can be used for later analysis. In each sample, the differences between groups of OTUs were recorded using PLS-DA. To obtain the species classification information corresponding to each OTU, the Ribosomal Database Project (RDP, version 2.2) classifier was used to perform taxonomic analysis on the representative sequence of OTU, and the community composition of each sample was calculated at the levels of phylum, family, genus, and species.

α- and β-diversity were estimated by MOTHUR (version 1.31.2) and QIIME (version 1.8.0), respectively, at the OTU level. For α-diversity analysis, the Chao indices and Shannon index curves were generated, and the ACE and Chao and Simpson and Shannon indices were calculated. Sample cluster was conducted by QIIME (version 1.8.0) based on UPGMA. KEGG and COG functions were predicted using PICRUSt software. Barplots and heatmaps of different classification levels were plotted with the R package version 3.4.1 and the R package “gplots,” respectively.

### Untargeted metabolomics analysis by LC-MS/MS.

Untargeted metabolomics of the stool samples by LC-MS/MS was performed by the Beijing Genomics Institute Company, Ltd., (Shenzhen, China) using a Waters 2D UPLC tandem Q Exactive HF high-resolution mass spectrometer (Milford, MA, USA), collecting data in positive and negative ion modes separately to improve metabolite coverage. The untargeted raw data (raw file) was imported into Compound Discoverer 3.0 (Thermo Fisher Scientific, Waltham, MA, USA) for data processing, including peak extraction, peak alignment, and compound identification. Metabolite identification was conducted against the BGI reference Library (BGI inhouse-developed standard library), mzCloud (Thermo Fisher Scientific, MA, USA), ChemSpider, Kyoto Encyclopedia of Genes and Genomes (KEGG), Human Metabolome Database (HMDB), and Lipidmaps Database. Date preprocessing, statistical analysis, metabolite classification annotation, and functional annotation were performed on the resulting normalized peak intensities using meta X software (44, 45). The multivariate raw data were dimensionally reduced by principal-component analysis to analyze the grouping, trend (similarity and difference within and between groups of samples), and outliers (whether there are abnormal samples) of the observed variables in the data set. Differential metabolites were screened using the VIP values of the first two principal components of the PLS-DA model, combined with the differential fold change obtained by univariate analysis and the results of the Student’s *t* test. Spearman correlation coefficient and sparse generalized canonical correlation analysis (sGCCA) were applied to recognize the correlations between perturbed intestinal microbiota and varied fecal metabolites (17).

### Ethics approval and consent to participate.

The study protocol was reviewed and approved by the Research Ethics Committee of Henan Agricultural University. All animals and experiments were handled in strict accordance with good laboratory animal practice according to the Animal Ethics Procedures and Guidelines of the People’s Republic of China. All efforts were made to minimize animal suffering.

### Data availability.

The 16S raw fastq sequencing files used within this study have been uploaded to the short reads archive (SRA) database (BioProjectID: PRJNA881406). The metabolomics data that support the findings of this study have been deposited into CNGB Sequence Archive (CNSA) of China National GeneBank DataBase (CNGBdb) with accession number CNP0003830.
