# Supplementary material for: Microbiome-Metabolomics Analysis of the Impacts of Cryptosporidium muris Infection in BALB/C Mice
Source: Microbiol Spectr. 2022 Dec 19;11(1):e02175-22. doi: 10.1128/spectrum.02175-22 (PMC9927150; doi:10.1128/spectrum.02175-22)
Supplement: Supplemental file 1 — Fig. S1 to S4 and Tables S1 to S3. Download spectrum.02175-22-s0001.pdf, PDF file, 1.2 MB [file spectrum.02175-22-s0001.pdf]

SUPPLEMENTAL MATERIAL

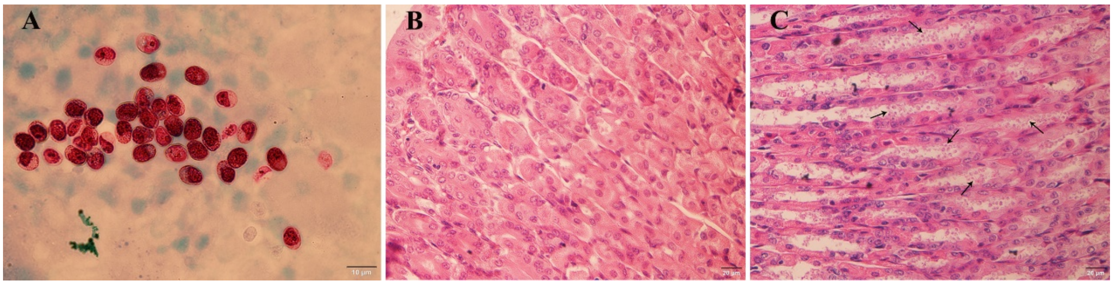

Figure S1 Observations of pathological sections of stomach tissue of BALB/c mice stained with modified acid-fast staining and hematoxylin and eosin staining. A: Oocyst of *C. muris* obtained from these mice under modified acid-fast microscopy. Bar = 10 μm; B, longitudinal section of gastric epithelium, negative control. C, gastric pits in stomach tissue sections of BALB/c mice infected with *C. muris* (arrow) which were sacrificed 14 days post-infection. A scale bar is included in each figure

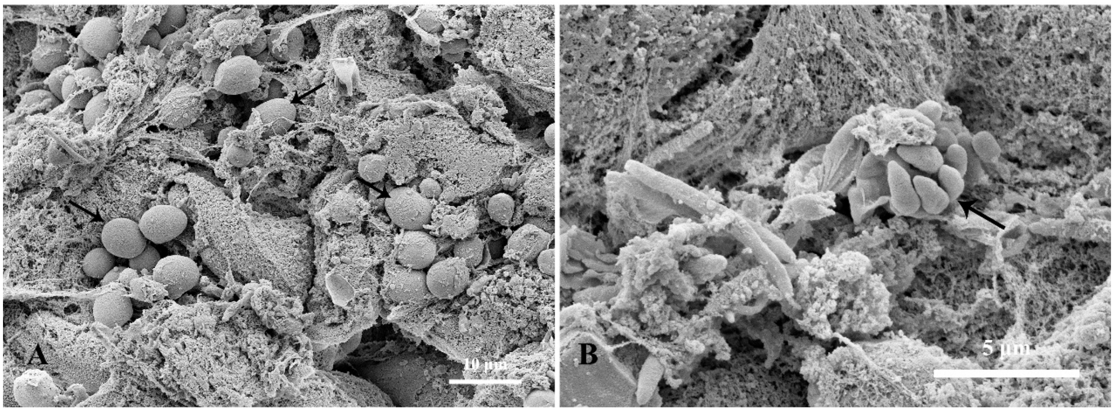

Figure S2 Scanning electron microscopy observation of stomach tissue after infection of BALB/c mice with 3×10^6 *C. muris* oocysts. *C. muris* (A, 5000×) and merozoites (B, 20000×) adhere to the surface of epithelial cells in the glandular portion of the gastric mucosa (arrow).

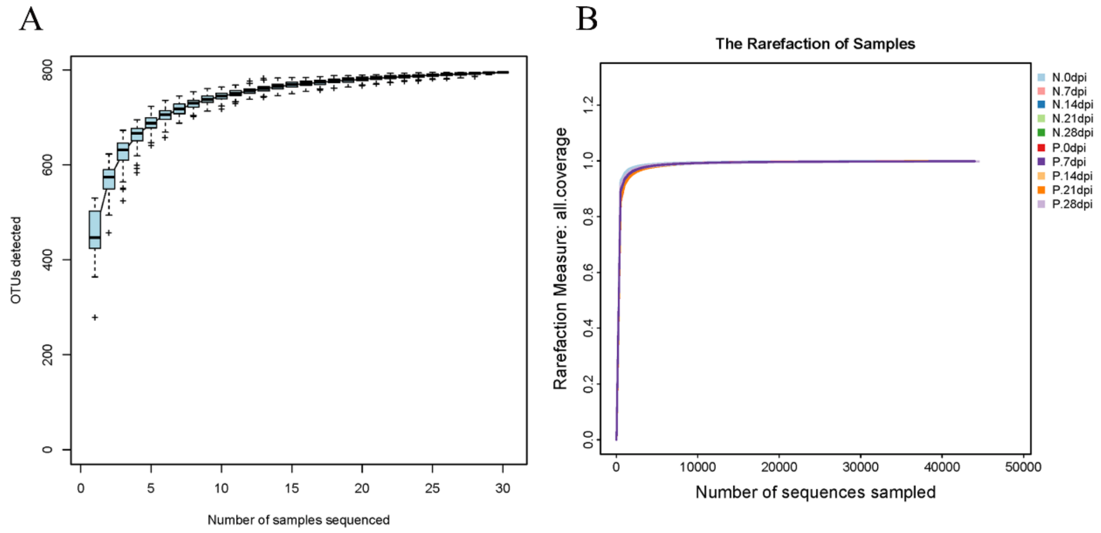

Figure S3 Species accumulation curves of all 30 fecal samples (A) and Rarefaction curves comparing the number of sequences with the number of phylotypes found in the 16S rRNA gene libraries from the microbiota in the fecal of the mice.

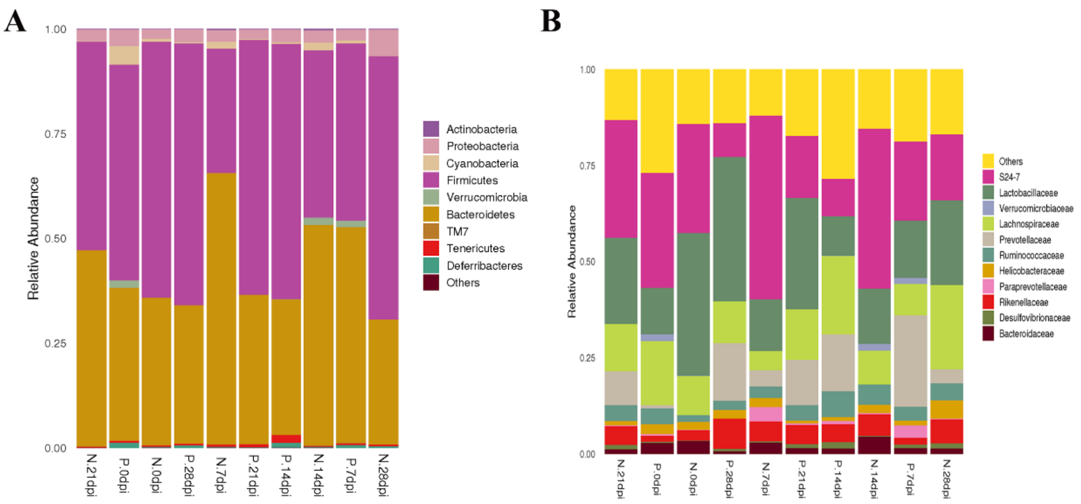

Figure S4 Comparison of the fecal-microbiota structures and distributions at the phylum (A) and family (B) levels of mice in infected and control groups. The abscissa is the sample name, and the ordinate is the relative abundance of the annotated species. The unannotated species of this classification level are classified as Unclassified Species with abundance below 0.5% in all samples are combined into Others.

Table S1. α-Diversity Indices of the Fecal Microbiota of the *C.muris* infected and control groups

Group	richness estimator				diversity index			
	ACE	*p* value	Chao	*p* value	Simpson	*p* value	Shannon	*p* value
N-0Dpi	413.90±94.41	0.130	424.71±89.73	0.138	3.67±0.61	0.77	0.088±0.05	0.139
P-0Dpi	544.50±5.46		550.00±22.09		4.54±0.18		0.025±0.007	
N-7Dpi	449.46±24.45	0.285	450.34±23.49	0.205	4.21±0.14	0.375	0.036±0.006	0.445
P-7Dpi	473.96±24.21		485.35±32.43		4.02±0.29		0.048±0.023	
N-14Dpi	488.97±24.51	0.008**	499.37±19.57	0.004**	4.103±0.048	0.161	0.041±0.003	0.580
P-14Dpi	560.54±5.26		573.13±9.18		4.523±0.337		0.032±0.024	
N-21Dpi	552.38±31.66	0.372	563.33±27.36	0.233	4.19±0.25	0.515	0.049±0.018	0.397
P-21Dpi	529.38±23.81		531.37±28.32		3.95±0.53		0.077±0.048	
N-28Dpi	501.37±34.57	0.282	508.16±25.87	0.282	4.15±0.31	0.130	0.051±0.033	0.211
P-28Dpi	452.76±58.33		462.80±57.64		3.44±0.56		0.112±0.063	

The values are represented as the means ± SD (n=3), the asterisk (* and **) represents the significant difference of $p < 0.05$ and $p < 0.01$, respectively, which determined by t-test.

Table S2 Summary of differential metabolites at different stages of infection in different ions

Mode	Group	Total number of differential metabolites	Number of differential metabolites with identified information	Up	Down
POS	N7 dpi: P7 dpi	602	325	343	259
	N14 dpi: P14 dpi	381	195	252	129
	N21 dpi: P21 dpi	328	165	238	90
	N28 dpi: P28 dpi	299	186	195	104
NEG	N7 dpi: P7 dpi	285	147	162	123
	N14 dpi: P14 dpi	261	117	145	116
	N21 dpi: P21 dpi	96	46	48	48
	N28 dpi: P28 dpi	93	48	39	54

Table S3. Differential pathways of differential metabolites in pos and neg mode at different infection stages by KEGG enrichment analysis

POS	7 DPI(31)	**Pathway.ID**	**Pathway**	**Count**	**P value**	**KEGG.Names**	**KEGG.IDs**
		map01100	Metabolic pathways	25	0.0000000001398865	Cytosine;L-citrulline; Uric acid; Uracil; Thymine; 1-methylxanthine; Pantothenic acid; 5'-s-methyl-5'-thioadenosine; 1,2-dihydroxy-3-keto-5-methylthiopentene; L-tryptophan; 4-hydroxybenzaldehyde;Biotin; Indole; Desthiobiotin; Cortisol; Corticosterone; Hydroxyprogesterone; Desoxycortone; Coproporphyrin; 2878846; 3,4-dihydroxyphenylacetic acid; (-)-shikimic acid; Phytosphingosine; Arachidonic acid; 8z,11z,14z-eicosatrienoic acid	C00380+C00327+C00366+C00106+C00178+C16358+C00864+C00170+C15606+C00078+C00633+C00120+C00463+C01909+C00735+C02140+C01176+C03205+C05769+C16678+C01161+C00493+C12144+C00219+C03242
		map00140	Steroid hormone	5	0.00002361937	(20s)-17,20-dihydroxypregn-4-en-3-	C04518+C00735+C02140+

			biosynthesis			one;<br>Cortisol;<br>Corticosterone;<br>Hydroxyprogesterone;<br>Desoxycortone	C01176+C03205
		map04925	Aldosterone synthesis and secretion	3	0.00005814966	Corticosterone;<br>Desoxycortone;<br>Arachidonic acid	C02140+C03205+C00219
		map00240	Pyrimidine metabolism	4	0.00007548624	Cytosine;<br>5-methylcytosine;<br>Uracil;<br>Thymine	C00380+C02376+C00106+C00178
		map01040	Biosynthesis of unsaturated fatty acids	4	0.000125333	Adrenic acid;<br>Eicosapentanoic acid;<br>Arachidonic acid;<br>8z,11z,14z-eicosatrienoic acid	C16527+C06428+C00219+C03242
		map00400	Phenylalanine, tyrosine and tryptophan biosynthesis	3	0.000239325	L-tryptophan;<br>Indole;(-)-shikimic acid	C00078+C00463+C00493
		map04927	Cortisol synthesis and secretion	2	0.000761536	Cortisol;<br>Hydroxyprogesterone	C00735+C01176
		map04923	Regulation of lipolysis in adipocytes	2	0.001045317	Corticosterone;<br>Arachidonic acid	C02140+C00219

		map04913	Ovarian steroidogenesis	2	0.003100502	Hydroxyprogesterone;<br>Arachidonic acid	C01176+C00219
		map00591	Linoleic acid metabolism	2	0.004208699	Arachidonic acid;<br>8z,11z,14z-eicosatrienoic acid	C00219+C03242
		map00770	Pantothenate and CoA biosynthesis	2	0.004208699	Uracil;Pantothenic acid	C00106+C00864
		map00780	Biotin metabolism	2	0.004208699	Biotin;<br>Desthiobiotin	C00120+C01909
		map04216	Ferroptosis	2	0.004510409	Adrenic acid;<br>Arachidonic acid	C16527+C00219
		map00410	beta-Alanine metabolism	2	0.00547362	Uracil;<br>Pantothenic acid	C00106+C00864
		map04977	Vitamin digestion and absorption	2	0.008051188	Pantothenic acid;<br>Biotin	C00864+C00120
		map04726	Serotonergic synapse	2	0.009292983	L-tryptophan;<br>Arachidonic acid	C00078+C00219
		map01230	Biosynthesis of amino acids	3	0.009935962	L-citrulline;<br>L-tryptophan;<br>(-)-shikimic acid	C00327+C00078+C00493
		map04974	Protein digestion and absorption	2	0.01153895	L-tryptophan;<br>Indole	C00078+C00463
		map00270	Cysteine and methionine metabolism	2	0.01893876	5'-s-methyl-5'-thioadenosine;<br>1,2-dihydroxy-3-keto-5-methylthiopentene	C00170+C15606

		map04912	GnRH signaling pathway	1	0.02059628	Arachidonic acid	C00219
		map04960	Aldosterone-regulated sodium reabsorption	1	0.02736846	Cortisol	C00735
		map04666	Fc gamma R-mediated phagocytosis	1	0.02736846	Arachidonic acid	C00219
		map00590	Arachidonic acid metabolism	2	0.02784652	Mfcd00065806;<br>Arachidonic acid	C14717+C00219
		map04730	Long-term depression	1	0.03073725	Arachidonic acid	C00219
		map00380	Tryptophan metabolism	2	0.03208691	L-tryptophan;<br>Indole	C00078+C00463
		map04217	Necroptosis	1	0.03409455	Arachidonic acid	C00219
		map04664	Fc epsilon RI signaling pathway	1	0.0374404	Arachidonic acid	C00219
		map04921	Oxytocin signaling pathway	1	0.04077484	Arachidonic acid	C00219
		map04728	Dopaminergic synapse	1	0.04077484	3,4-dihydroxyphenylacetic acid	C01161
		map04976	Bile secretion	2	0.04452182	Uric acid;<br>Cortisol	C00366+C00735

		map04611	Platelet activation	1	0.04740965	Arachidonic acid	C00219
	14 DPI(31)	map01100	Metabolic pathways	15	0.0000001007892	Choline;<br>L-serine o-phosphate;Taurine;<br>Acpc;<br>L-glutamic acid;<br>L-pyroglutamic acid;<br>N-acetyl-l-aspartic acid;<br>7-aminomethyl-7-deazaguanine;<br>Melatonin;<br>Desthiobiotin;<br>Cholic acid;<br>4,4-dimethyl-5alpha-cholesta-8,14,24-trien-3beta-ol;<br>P-xylene;P-cresol;Phylloquinone oxide	C00114+C01005+C00245+C01234+C00025+C01879+C01042+C16675+C01598+C01909+C00695+C11455+C06756+C01468+C05849
		map04976	Bile secretion	4	0.0000292751	Choline;<br>Taurochenodeoxycholic acid;<br>Cholic acid;<br>Deoxycholate	C00114+C05465+C00695+C04483
		map00120	Primary bile acid biosynthesis	3	0.00008721079	Taurine;<br>Taurochenodeoxycholic acid;<br>Cholic acid	C00245+C05465+C00695
		map04713	Circadian entrainment	2	0.000117253	L-glutamic acid;<br>Melatonin	C00025+C01598
		map04080	Neuroactive	3	0.000118118	Taurine;L-glutamic acid;	C00245+C00025+C01598

			ligand-receptor interaction			Melatonin	
		map00430	Taurine and hypotaurine metabolism	2	0.000741177	Taurine;<br>L-glutamic acid	C00245+C00025
		map00250	Alanine, aspartate and glutamate metabolism	2	0.00120448	L-glutamic acid;<br>N-acetyl-l-aspartic acid	C00025+C01042
		map02010	ABC transporters	3	0.001585518	Choline;<br>Taurine;<br>L-glutamic acid	C00114+C00245+C00025
		map00480	Glutathione metabolism	2	0.002214439	L-glutamic acid;<br>L-pyroglutamic acid	C00025+C01879
		map04974	Protein digestion and absorption	2	0.003370073	L-glutamic acid;<br>P-cresol	C00025+C01468
		map00340	Histidine metabolism	2	0.003370073	Carglumic acid;<br>L-glutamic acid	C05829+C00025
		map00260	Glycine, serine and threonine metabolism	2	0.003805856	Choline;<br>L-serine o-phosphate	C00114+C01005
		map00970	Aminoacyl-tRNA biosynthesis	2	0.00411019	L-serine o-phosphate;<br>L-glutamic acid	C01005+C00025
		map00270	Cysteine and methionine	2	0.005614116	L-serine o-phosphate;<br>Acpc	C01005+C01234

			metabolism				
		map04068	FoxO signaling pathway	1	0.009163371	L-glutamic acid	C00025
		map04720	Long-term potentiation	1	0.01280585	L-glutamic acid	C00025
		map04724	Glutamatergic synapse	1	0.01462222	L-glutamic acid	C00025
		map04730	Long-term depression	1	0.01643534	L-glutamic acid	C00025
		map04727	GABAergic synapse	1	0.01643534	L-glutamic acid	C00025
		map01200	Carbon metabolism	2	0.01798837	L-serine o-phosphate; L-glutamic acid	C01005+C00025
		map04979	Cholesterol metabolism	1	0.01824522	Taurochenodeoxycholic acid	C05465
		map04540	Gap junction	1	0.02005187	L-glutamic acid	C00025
		map04072	Phospholipase D signaling pathway	1	0.02005187	L-glutamic acid	C00025
		map00471	D-Glutamine and D-glutamate metabolism	1	0.02185529	L-glutamic acid	C00025
		map04725	Cholinergic synapse	1	0.02185529	Choline	C00114
		map04721	Synaptic vesicle cycle	1	0.02185529	L-glutamic acid	C00025

		map01230	Biosynthesis of amino acids	2	0.02309624	L-serine o-phosphate;<br>L-glutamic acid	C01005+C00025
		map04964	Proximal tubule bicarbonate reclamation	1	0.0308242	L-glutamic acid	C00025
		map04723	Retrograde endocannabinoid signaling	1	0.03438937	L-glutamic acid	C00025
		map00910	Nitrogen metabolism	1	0.03438937	L-glutamic acid	C00025
		map00220	Arginine biosynthesis	1	0.04148155	L-glutamic acid	C00025
	21 DPI(17)	map01100	Metabolic pathways	8	0.001814962	L-histidinol;D-(+)-glucosamine;<br>N-acetylmuramic acid;<br>L-phenylalanine;Serotonin;<br>Desthiobiotin;Corticosterone;<br>Cholic acid	C00860+C00329+C02713+<br>C00079+C00780+C01909+<br>C02140+C00695
		map04974	Protein digestion and absorption	2	0.001977779	2908;L-phenylalanine	C01746+C00079
		map00380	Tryptophan metabolism	2	0.005756575	Serotonin;<br>6-hydroxymelatonin	C00780+C05643
		map04976	Bile secretion	2	0.008159258	Serotonin;<br>Cholic acid	C00780+C00695
		map00140	Steroid hormone biosynthesis	2	0.008486367	(20s)-17,20-dihydroxypregn-4-en-3-one;<br>Corticosterone	C04518+C02140

		map00520	Amino sugar and nucleotide sugar metabolism	2	0.01003014	D-(+)-glucosamine; N-acetylmuramic acid	C00329+C02713
		map01230	Biosynthesis of amino acids	2	0.01386922	L-histidinol; L-phenylalanine	C00860+C00079
		map04540	Gap junction	1	0.01536695	Serotonin	C00780
		map04721	Synaptic vesicle cycle	1	0.01675264	Serotonin	C00780
		map04923	Regulation of lipolysis in adipocytes	1	0.01951837	Corticosterone	C02140
		map04925	Aldosterone synthesis and secretion	1	0.03050671	Corticosterone	C02140
		map04024	cAMP signaling pathway	1	0.03459673	Serotonin	C00780
		map00780	Biotin metabolism	1	0.03867016	Desthiobiotin	C01909
		map04978	Mineral absorption	1	0.04002429	L-phenylalanine	C00079
		map04742	Taste transduction	1	0.04407569	Serotonin	C00780
		map04750	Inflammatory mediator regulation of	1	0.04811065	Serotonin	C00780

			TRP channels				
		map00400	Phenylalanine, tyrosine and tryptophan biosynthesis	1	0.04811065	L-phenylalanine	C00079
	28 DPI(7)	map04713	Circadian entrainment	1	0.01210835	Melatonin	C01598
		map04728	Dopaminergic synapse	1	0.01611309	3,4-dihydroxyphenylacetic acid	C01161
		map04071	Sphingolipid signaling pathway	1	0.02010225	Sphinganine	C00836
		map01100	Metabolic pathways	6	0.02380746	Spermidine;N-acetylornithine; Melatonin;Sinapinic acid; Sphinganine; 3,4-dihydroxyphenylacetic acid	C00315+C00437+C01598+ C00482+C00836+C01161
		map00220	Arginine biosynthesis	1	0.03066423	N-acetylornithine	C00437
		map00600	Sphingolipid metabolism	1	0.0332876	Sphinganine	C00836
		map00410	beta-Alanine metabolism	1	0.04241583	Spermidine	C00315
NEG	7 DPI(22)	map01040	Biosynthesis of unsaturated fatty acids	4	0.000001344588	8z,11z,14z-eicosatrienoic acid; Adrenic acid; 11(z),14(z)-eicosadienoic acid; Arachidonic acid	C03242+C16527+C16525+ C00219

		map04270	Vascular smooth muscle contraction	2	0.000146084	11,12-epoxy-(5z,8z,11z)-icosatrienoic acid;<br>Arachidonic acid	C14770+C00219
		map04913	Ovarian steroidogenesis	2	0.000334156	11,12-epoxy-(5z,8z,11z)-icosatrienoic acid;<br>Arachidonic acid	C14770+C00219
		map00591	Linoleic acid metabolism	2	0.000456397	8z,11z,14z-eicosatrienoic acid;<br>Arachidonic acid	C03242+C00219
		map04216	Ferroptosis	2	0.000489868	Adrenic acid;<br>Arachidonic acid	C16527+C00219
		map00860	Porphyrin and chlorophyll metabolism	3	0.00053326	Urobilinogen;Stercobilin;<br>Protoporphyrinogen	C05790+C05793+C01079
		map04726	Serotonergic synapse	2	0.001029656	11,12-epoxy-(5z,8z,11z)-icosatrienoic acid;<br>Arachidonic acid	C14770+C00219
		map00590	Arachidonic acid metabolism	2	0.003244536	11,12-epoxy-(5z,8z,11z)-icosatrienoic acid;<br>Arachidonic acid	C14770+C00219
		map04912	GnRH signaling pathway	1	0.006797558	Arachidonic acid	C00219
		map04666	Fc gamma R-mediated phagocytosis	1	0.009053614	Arachidonic acid	C00219
		map01100	Metabolic pathways	6	0.01003086	Uric acid;<br>2-aminoadipic acid;	C00366+C00956+C03242+<br>C01079+C14770+C00219

						8z,11z,14z-eicosatrienoic acid; Protoporphyrinogen; 11,12-epoxy-(5z,8z,11z)-icosatrienoic acid; Arachidonic acid	
		map04730	Long-term depression	1	0.01017981	Arachidonic acid	C00219
		map04217	Necroptosis	1	0.01130479	Arachidonic acid	C00219
		map04664	Fc epsilon RI signaling pathway	1	0.01242855	Arachidonic acid	C00219
		map04921	Oxytocin signaling pathway	1	0.01355109	Arachidonic acid	C00219
		map04611	Platelet activation	1	0.01579254	Arachidonic acid	C00219
		map04923	Regulation of lipolysis in adipocytes	1	0.01579254	Arachidonic acid	C00219
		map04723	Retrograde endocannabinoid signaling	1	0.02137494	Arachidonic acid	C00219
		map04925	Aldosterone synthesis and secretion	1	0.0247099	Arachidonic acid	C00219
		map00600	Sphingolipid	1	0.02803403	Mfcd00871363	C01120

			metabolism				
		map00410	beta-Alanine metabolism	1	0.03574842	Malonate	C00383
		map04750	Inflammatory mediator regulation of TRP channels	1	0.03903671	Arachidonic acid	C00219
	14 DPI(26)	map00120	Primary bile acid biosynthesis	3	0.000006859809	Taurine; Taurochenodeoxycholic acid; Chenodeoxycholate	C00245+C05465+C02528
		map01100	Metabolic pathways	8	0.00001889142	Oxoglutaric acid; N-acetylaspartic acid; Taurine;N-acetylneuraminate; B-alanine; 8z,11z,14z-eicosatrienoic acid; Protoporphyrinogen; Chenodeoxycholate	C00026+C01042+C00245+ C00270+C00099+C03242+ C01079+C02528
		map04976	Bile secretion	3	0.00006087326	Oxoglutaric acid; Taurochenodeoxycholic acid; Chenodeoxycholate	C00026+C05465+C02528
		map00430	Taurine and hypotaurine metabolism	2	0.000140636	Oxoglutaric acid; Taurine	C00026+C00245
		map00250	Alanine, aspartate and glutamate	2	0.000229485	Oxoglutaric acid; N-acetylaspartic acid	C00026+C01042

			metabolism				
		map04080	Neuroactive ligand-receptor interaction	2	0.000796015	Taurine;<br>B-alanine	C00245+C00099
		map04727	GABAergic synapse	1	0.007280736	Oxoglutaric acid	C00026
		map04979	Cholesterol metabolism	1	0.008086646	Taurochenodeoxycholic acid	C05465
		map00471	D-Glutamine and D-glutamate metabolism	1	0.009696633	Oxoglutaric acid	C00026
		map04066	HIF-1 signaling pathway	1	0.01210704	Oxoglutaric acid	C00026
		map04964	Proximal tubule bicarbonate reclamation	1	0.01371094	Oxoglutaric acid	C00026
		map00020	Citrate cycle (TCA cycle)	1	0.01611222	Oxoglutaric acid	C00026
		map00220	Arginine biosynthesis	1	0.01850805	Oxoglutaric acid	C00026
		map04922	Glucagon signaling pathway	1	0.02089844	Oxoglutaric acid	C00026
		map00591	Linoleic acid metabolism	1	0.02248901	8z,11z,14z-eicosatrienoic acid	C03242
		map00770	Pantothenate	1	0.02248901	B-alanine	C00099

			and CoA biosynthesis				
		map00410	beta-Alanine metabolism	1	0.02566291	B-alanine	C00099
		map00920	Sulfur metabolism	1	0.02645489	Taurine	C00245
		map00650	Butanoate metabolism	1	0.03355565	Oxoglutaric acid	C00026
		map04974	Protein digestion and absorption	1	0.0374796	B-alanine	C00099
		map00340	Histidine metabolism	1	0.0374796	Oxoglutaric acid	C00026
		map00640	Propanoate metabolism	1	0.0382626	B-alanine	C00099
		map00053	Ascorbate and aldarate metabolism	1	0.03904501	Oxoglutaric acid	C00026
		map00310	Lysine degradation	1	0.04372701	Oxoglutaric acid	C00026
		map00040	Pentose and glucuronate interconversions	1	0.04372701	Oxoglutaric acid	C00026
		map00630	Glyoxylate and dicarboxylate metabolism	1	0.04916242	Oxoglutaric acid	C00026
	21	map04916	Melanogenesis	1	0.002594102	L-tyrosine	C00082

	DPI(13)	map04917	Prolactin signaling pathway	1	0.004751355	L-tyrosine	C00082
		map04728	Dopaminergic synapse	1	0.005182315	L-tyrosine	C00082
		map00730	Thiamine metabolism	1	0.01333959	L-tyrosine	C00082
		map00400	Phenylalanine, tyrosine and tryptophan biosynthesis	1	0.01504944	L-tyrosine	C00082
		map04974	Protein digestion and absorption	1	0.02016342	L-tyrosine	C00082
		map00970	Aminoacyl-tRNA biosynthesis	1	0.02228738	L-tyrosine	C00082
		map00360	Phenylalanine metabolism	1	0.030743	L-tyrosine	C00082
		map01100	Metabolic pathways	3	0.03111975	N-acetylmuramic acid; 4687;L-tyrosine	C02713+C00294+C00082
		map00350	Tyrosine metabolism	1	0.03326717	L-tyrosine	C00082
		map00130	Ubiquinone and other terpenoid-quinone biosynthesis	1	0.03913455	L-tyrosine	C00082

		map00230	Purine metabolism	1	0.04038779	4687	C00294
		map00520	Amino sugar and nucleotide sugar metabolism	1	0.04580196	N-acetylmuramic acid	C02713
	28 DPI(3)	map04216	Ferroptosis	1	0.006260775	1-stearoyl-sn-glycero-3-phosphoethanolamine	C21484
		map00410	beta-Alanine metabolism	1	0.00690676	Malonate	C00383
		map00240	Pyrimidine metabolism	1	0.01399182	Malonate	C00383
